# An Artemisinin Derivative of Praziquantel as an Orally Active Antischistosomal Agent

**DOI:** 10.1371/journal.pone.0112163

**Published:** 2014-11-11

**Authors:** Lanlan Dong, Wenwen Duan, Jinglei Chen, Huan Sun, Chunhua Qiao, Chao-ming Xia

**Affiliations:** College of Medical Science, Soochow University, Suzhou, China; UMASS Medical School, United States of America

## Abstract

**Background:**

Schistosomiasis is a major health problem in tropical and sub-tropical areas caused by species of trematode belonging to the genus *Schistosoma*. The treatment and control of this disease has been relying on the use of a single drug praziquantel. However, the drug resistance concern urged the development of new drugs against schistosoma. Here, we report our systematic biological evaluation of DW-3-15, a new lead compound developed based on our conjugation design rationale as an effective anti-schistosomal agent.

**Methodology/Principal Findings:**

The antischistosomal activity of DW-3-15 was systematically evaluated in *S. japonicum* infected mouse model for its stage-sensitivity and dose response. The results revealed that DW-3-15 exhibited 60–85% worm reduction rate against different development stage of worm. Scanning electron microscopy (SEM) observation indicated that DW-3-15 may damage to the tegument of male schistosomes.

**Conclusions/Significance:**

Our results demonstrated that DW-3-15 showed potent anti-schistosomal activities in vivo. The results strongly support our conjugation design strategy of artemisinin analogs and further development of DW-3-15 as a new lead compound as anti-schistosomal agent.

## Introduction

Schistosomias is a major health problem in the tropical and sub-tropical areas worldwide, particularly in the rural areas of sub-Saharan Africa [1]. Blood flukes of the genus *Schistosoma* is the cause of this diseas [2]. It is estimated that approximately 779 million people are at risk of schistosomiasis and over 200 million are infected with this disease, among which 20 million suffer from severe disease manifestations, i.e., chronic hepatic and intestinal fibrosis, ureteric and bladder fibrosis, and calcification of the genitourinary trac [3], [4].

**Figure 1:**
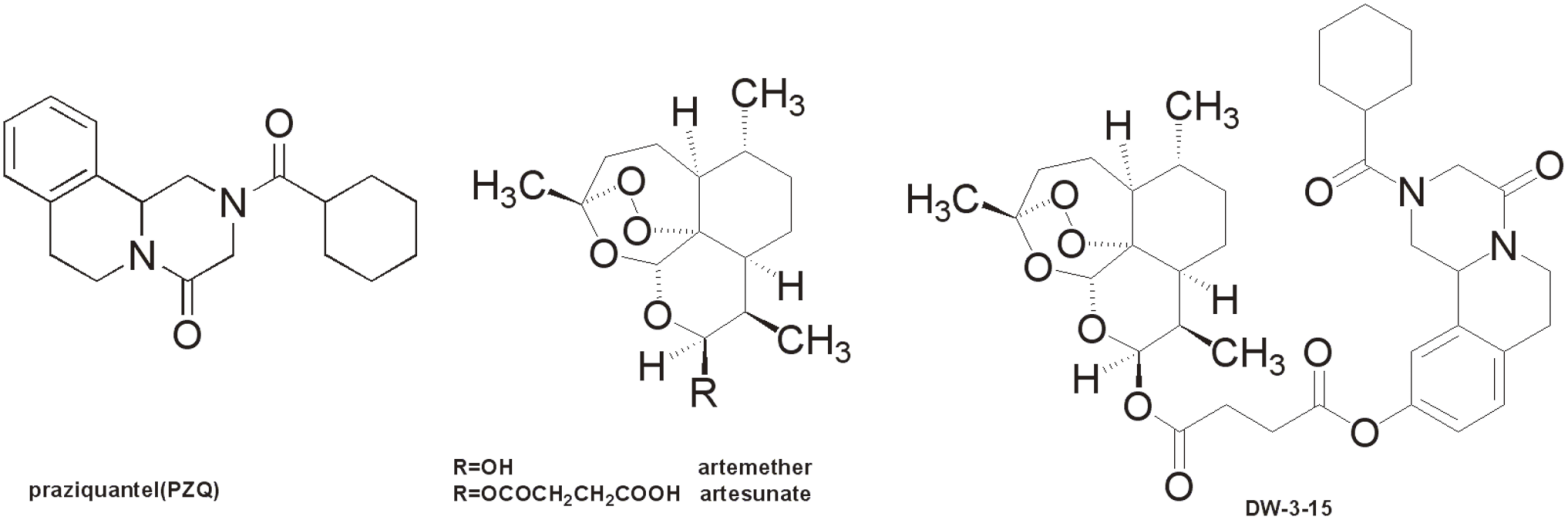
Structures of praziquental, artemether, artesunate and rationally designed DW-3-15.

Despite the high level of prevalence of this tropical disease, praziquantel (PZQ, Figure 1) is the only drug of choice for schistosomiasis control. After 40 years of implementation, low sensitivity to PZQ has been identified and reported recentl [5], [6]. PZQ undergoes rapid first-pass drug metabolism to give a significantly less potent metabolit [7], [8]. PZQ also suffers from low efficacy against juvenile forms of schistosome [9], [10]. Hence, multiple doses are necessary to remove those parasites that have since matured after initial exposure. Nevertheless, the dependence of this single drug to treat a disease that affects hundred millions of people is not sustainable.

Efforts to pursue novel antischistosomal drugs have identified a number of compounds with promising antischistosomal properties. Targeting a parasite enzyme thioredoxin glutathione reductase (TGR), a quantitative high–throughput screening method has selected oxadiazole 2-oxides as new lead compounds with significant worm burden reduction activit [1]. A cysteine protease inhibitor K11777 could significantly reduce egg production and worm burden in *S. mansoni* infected mice mode [11]. An unsaturated fatty acid―arachidonic acid was reported to lead obvious decrease in the total worm burden through activation of parasite tegument-bound neutral sphingomyelinase (nSMase) [12]. Along with these molecular target specific agents, other chemotherapeutic chemicals with uncertain mechanism of actions were also reported. For example, revisit of an old anti-malarial drug mefloquine has revealed its promising anti-schistosomal properties in mice mode [13]; a cyclic nucleotide analogue 9-(S)-[3-hydroxy-2-(phosphonomethoxy)propyl] adenine [(S)-HPMPA] was able to induce significant decrease in the total worm burde [14]. Likewise, chemical modification on PZQ structure to some organometallic-containing PZQ derivatives has identified the discovery of novel active compounds against schistosomiasi [15], [16]. However, the paucity of available biological resource for schistosomiasis and other neglected tropical diseases has hampered the drug discovery and development process.

Originally discovered as an effective antimalarial drug, artemisinin analogs such as artemether and artesunate (ARTs, Figure 1) have also been revealed to show maximal activities against young developmental stages of the schistosomes. It was reported that artemisinin and its analogs eradicate *Plasmodium* via a radical-mediated cytotoxic mechanism by targeting the redox active iron-heme comple [17]. Since both *Plasmodium* and *Schistosoma* parasites share the same iron-heme mechanism, artemisinin derivatives have been demonstrated cytotoxic effects against *Schistosoma* patients infected with all three species of schistosomes: *S. mansoni*, *S. haematobium* and *S. japonicu* [18].

The complementary effects of PZQ and artemisinin against schistosomes have been recognized recently. PZQ is highly effective against the adult stage of schistosomes but less effective against young developing stage of schistosomul [19], while artemisinin is more effective against schistosomule [20]. It is anticipated that conjugation of both molecules would possess potential broad-spectrum antischistosomal activity against both juvenile and adult forms of schistosomes. The same design rational was employed by Julien et al for a trioxaquine molecule named PA1259, which is a hybrid drug containing a trioxane and an aminoquinoline pharmacophore with activity both in vitro and in vivo against all schistosome stages (cercariae, schistosomule and adult) [21]. PZQ conjugates with synthetic peroxide moieties connected at the PZQ metabolism liable position were synthesized and their anti-schistosomal activity was reporte [22]. Unfortunately, none of these compounds displayed efficient worm killing activity.

Based on the same design rationale, we have prepared PZQ conjugates with linkage at position-10 of the PZQ scaffold, and this has led to the discovery of one promising lead compoun [23], DW-3-15 (Figure1), that exhibited good worm killing activity both *in* *vitro* and *in* *vivo*. Herein, we report the continuing effort to investigate DW-3-15 as a novel anti-schistosomal drug candidate.

## Materials and Methods

### Ethics statement

All animal study was conducted in compliance with the Regulations for the Care and Use of Laboratory Animals, and the protocol was approved by the committee on the ethics of animal experimentation of the Soochow University (Permit Number: 2007-13).

### Drugs

DW-3-15 was prepared according to the previously reported metho [23]. The purity was confirmed to be >95% by HPLC analysis (80% MeOH, 20% H_2_O, C18 column). The solutions of DW-3-15 were freshly prepared in homogenous aqueous suspensions containing 7% Tween 80 and 3% ethanol. Artesunate and PZQ were purchased from Sinopharm Chemical Reagent Co. Ltd. (China).

### Parasites

*S. japonicum* cercariae (Anhui isolate), released from infected intermediate host snail *Oncomelania hupensis,* was provided by the Institute of Schistosomiasis Control in Jiangsu Province (Wuxi, China).

### Animals and Experimental Infection

Female ICR strain mice (4–6 weeks old, 20∼22 g), provided by the Experimental Animal Center of Soochow University, were maintained under environmentally controlled conditions: 25°C, 12 h light and 12 h dark cycle, free access to water and rodent food. Mice were acclimatized for 5–7 days prior to infections. Method for mouse infection and parasite recovery were performed according to the previously described procedur [24]. To minimize suffering, mice were euthanized with peritoneal (i.p.) injections of 50 mg/kg sodium pentobarbital before collecting worms.

### Activity of DW-3-15 on mice infected with *S. japonicum*

Mice were infected with *S. japonicum* cercariae (∼60/mouse) via shaved abdominal skin. Then, infected mice were randomly grouped (10 mice/group). Mice were treated orally with DW-3-15 or PZQ or artesunate. At different stages of postinfection, mice were treated with DW-3-15 at a single oral dose of 100–400 mg/kg for 5 consecutive days, the specific dosage was indicated for each experiment in the following text. Animals were sacrificed on the 21st day of post-treatment by blood letting, and *S. japonicum* was recovered from the hepatic and portomesenteric veins by perfusion. The number of seized worm and worm reduction rate were recorded and calculated, respectivel [25].

For understanding the effect of DW-3-15 on juvenile and adult schistosomes harbored in the same host, mice were initially infected with 30 schistosome cercariae, followed by the second infection with the same number of the cercariae 42 days post the first infection. DW-3-15 was administered orally at 200 mg/kg on day 1, 3, 7, 14 and 21 after the second infection for 5 consecutive days. After 21 days, mice were sacrificed and worms were recovered from the hepatic and mesenteric veins and counted.

### Scanning Electron Microscopy (SEM) study of DW-3-15 damaged Schistosomes

Schistosomes were cultured in Dulbecco’s modified minimum Eagle’s medium (DMEM) supplemented with 20% newborn calf serum, 100 U mL^−1^ penicillin, 100 µg mL^−1^ streptomycin and 0.5 µg mL^−1^ amphotericin B. DW-3-15 (25 µM, 17.4 µg/mL) was added and the culture dish (4.0 mL) was kept at 37 °C in 95% air+5% CO_2_ for 72 h. The schistosomes were then washed with saline (3×500 µL), fixed in 4% glutaraldehyde-PBS buffer (0.1 M, pH 7.4) overnight, again washed with the same PBS buffer twice, followed by fixation with 1% OsO_4_ for 1 hour. After dehydration with ethanol, they were mounted on aluminum stubs, followed by platinum sputtering during 160 seconds. The control worms were simultaneously processed without drug treatment. For each group, two male worms were examined.

To investigate the drug effect on worm tegument *in* *vivo*, mice were fed with 200 mg/kg DW-3-15 on day 35 of infection, and schistosomes were recovered after 24 h. The collected worms were treated as above, and SEM images were recorded. We used the EDAX S570 SEM-FEG microscope with an accelerating voltage of 30 kV. The body of schistosomes was located, and photographs were then recorded with magnification x3000.

### Statistical Analysis

All data sets were analyzed using the SPSS16.0 software package. Statistical significance of the difference of two sets of data and the sample rates was determined by the t-test and the chi-square test, respectively. A difference in median was considered to be significant at 5% or less.

## Results

### Stage-sensitivity of DW-3-15 *in* *vivo*

**Figure 2:**
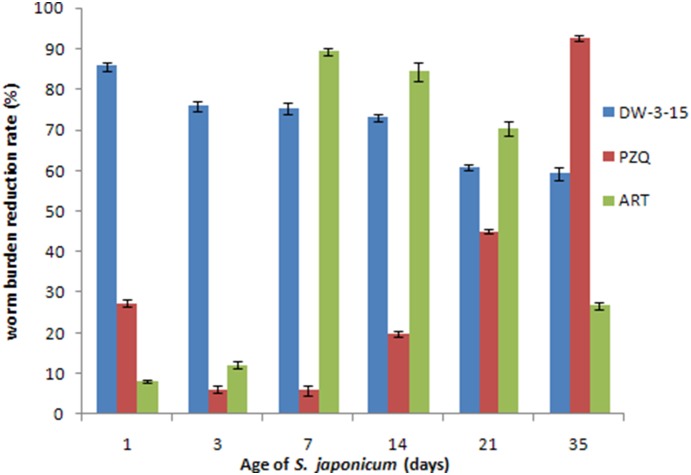
Susceptibility of various stages of Schistosoma japonica to DW-3-15, praziquantel (PZQ) and artesunate at a daily dose of 200 mg/kg for 5 days in mice. Blue: DW-3-15; Red: PZQ; Green: ART. *p-value<0.05.

As shown in Figure 2, significant worm reduction rate (greater than 70%) was observed for DW-3-15 when administered before day 14 postinfection. Notably, a high worm burden reduction rate of 85.7% was observed on day 1 of postinfection, compared to the 27.2% worm reduction rate for PZQ and 8.9% for artesunate. On day 3 postinfection, DW-3-15 is able to reduce the worm burden by >75%, both PZQ and artesunate are slightly active. On day 7, the activity of DW-3-15 remains highly efficient compared to PZQ, artesunate started to show significant worm killing capability. Even on day 14, DW-3-15 is significantly more active than PZQ, the worm reduction rate is 70.5% for DW-3-15 and 19.8% for PZQ, respectively. On day 21, the effect of DW-3-15 drop to 60.9%, while 45% worm burden reduction for PZQ was observed. On day 35, worm adult stage, the total worm burden reduction rate for PZQ is 86.7%, which is higher than that of 59.2% from DW-3-15. Overall, artesunate was highly efficient between day 7 and day 14, and started to show low worm reducing ability at the adult worm stage (day 35 with worm reducing rate of 26.8% only).

### The dose-response of DW-3-15 against adult schistosomes

In view of the promising antischistosomal activity of DW-3-15, we further test its ability against the adult worm. As shown in Table 1, a moderate total worm burden reduction rate of 40% was achieved at 100-mg/kg for 5 consecutive days. The reduction rate was increased to 56.2% at 200 mg/kg. Further dosage increase to 400 mg/kg did not significantly improve the reduction rate, with a 58.3% rate being observed.

**Table 1:** The dose-dependent effect of DW-3-15 in oral treatment of mice infected with adult (35-day-old) *Schistosoma japonicum* at a daily dose of 100–400 mg/kg for 5 days.

dosage (mg/kg per day × day)	control	100×5	200×5	400×5
Number of mice tested	10	12	12	12
Total worm burden 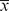 ± *s*)	43.0±4.0	25.7±6.6	18.4±4.3	17.5±5.1
Total worm reduction (%)	0	40.0*	56.2*	58.3*

*p-value<0.05.

### Comparison of DW-3-15 with artesunate against juvenile worm in mice model

Table 2 summarizes the activity of DW-3-15 against the 14-day juvenile worm harbored in ICR mice. Drugs were administrated orally at 200 mg/kg for 5 consecutive days. A total worm reduction of 74.3% was observed for DW-3-15 after administration. This rate is comparable to that of artesunate (83.3%, dosage 5×200 mg/kg) but significantly higher than that of PZQ (16.7%, dosage 5×200 mg/kg).

**Table 2:** Effect of DW-3-15, artesunate and PZQ given orally to mice infected with 14-day-old *Schistosoma japonicum* at a daily dose of 200 mg/kg for 5 days.

Compound	Number of Mice tested	Total worm burden ( 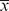 ± *s*)	Total worm reduction (%)
Control	10	40.8±6.8	0
DW-3-15	12	10.5±4.2	74.3**
Artesunate	12	6.8±3.0	83.3**
PZQ	12	34.0±6.6	16.2

**p-value<0.005.

### Effect of DW-3-15 on mice simultaneously harboring with juvenile and adult schistosomes

To further demonstrate the efficacy of DW-3-15 against various stages of schistosomes harbored in the same host, the drug activity was evaluated using a twice infected mouse model in which the mice were harbored both juvenile and adult worms. This animal model was designed to mimic the real world societ [26].

Table 3 shows the results obtained after administration of DW-3-15 to mice bearing different development stage of *S. japonicum*. On day 1 of re-infection, the mice harbor 42 day adult worm and 1 day schistosomules in the body, significant worm burden reduction rate (83.9%) was observed. In the following 3–14 days, the mice harbor both adult and juvenile worm in the body, the activity of DW-3-15 was demonstrated by exhibiting modest to high worm reduction rate (65.3% on day 3, 68.8% on day 7, and 70.5% on day 14). As control, administration of the same dosage of PZQ yields very low worm reduction rates. Especially, PZQ exhibited lower than 10.0% worm reduction from day 3 to day 14 time window. On day 21, the total worm reduction rate for DW-3-15 was 60.4%, this number is significantly higher than that observed for PZQ (10.9%). For artesunate, high worm reduction rate was observed between day 7 and day 21, and the rate decreased tremendously to 28.8% when all worm grown adult (day 35).

**Table 3:** Effect of DW-3-15 given at a daily dose of 200 mg/kg for 5 days to mice harboring simultaneously with 43–64 day-old adult and d1, d3, d7, d14, or d21 day old juvenile *Schistosoma japonicum*.

	d 1		d 3		d 7		d 14		d 21		d 42	
GroupWorm age	Totalwormburden(*x* ± *s*)	Totalwormreduction (%)(*x* ± *s*)	Totalwormburden	Totalwormreduction (%)(*x* ± *s*)	Totalwormburden(*x* ± *s*)	Totalwormreduction (%)(*x* ± *s*)	Totalwormburden(*x* ± *s*)	Totalwormreduction (%)(*x* ± *s*)	Totalwormburden(*x* ± *s*)	Totalwormreduction (%)(*x* ± *s*)	Totalwormburden(*x* ± *s*)	Totalwormreduction (%)(*x* ± *s*)
DW-3-15	6.5±2.4	83.9**	14.0±3.3	65.3*	12.6±4.4	68.8*	11.9±3.4	70.5**	16.0±5.5	60.4*	16.0±4.9	60.2*
PZQ	29.0±5.3	28.2	38.4±4.0	4.9	37.2±2.5	8.0	37.8±3.1	6.4	36.0±4.0	10.9	3.1±2.6	92.1**
Artesunate	38.8±6.5	3.9	35.3±4.8	12.6	5.5±4.3	86.5**	4.7±3.5	88.3**	15.8±4.2	60.8*	28.8±3.8	28.8
Control	40.4±3.6		40.4±3.6		40.4±3.6		40.4±3.6		40.4±3.6		40.4±3.6	

*p-value<0.05,

**p-value<0.005.

Note: ten mice were used in each group.

### Mechanistic studies using Scanning Electron Microscopy (SEM)

**Figure 3:**
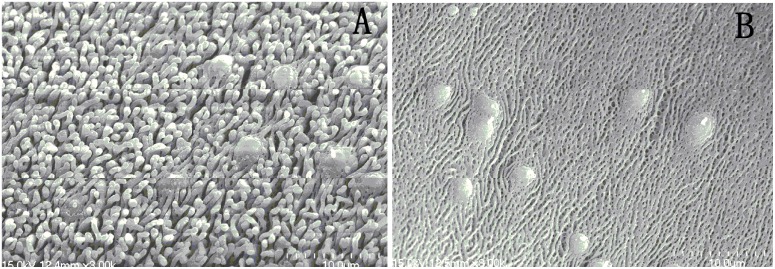
Scanning electron microscopy (SEM, ×3000) observation on the tegument of male adult *Schistosoma japonicum* harbored in control mice. A: tegumental ridges and sensory structures along dorsal (or ventral) part in mid-portion of the worm body (×3000); B: The tegument in the inner wall of the worm gynecophoral canal (×3000).

The normal morphology of worms was displayed in Figure 3. Figure 3A shows the mid-portion of a male worm body wall, 3B shows the inner wall of the gynecophoral canal. The general morphology of the mid-portion part of the worm exhibit even appearance with the ciliated sensory papillaes well distributed (Figure 3A); The tegument of the inner wall of gynecophoral canal of male worm shown regular clefts, the ridge was clearly presented (Figure 3B).

**Figure 4:**
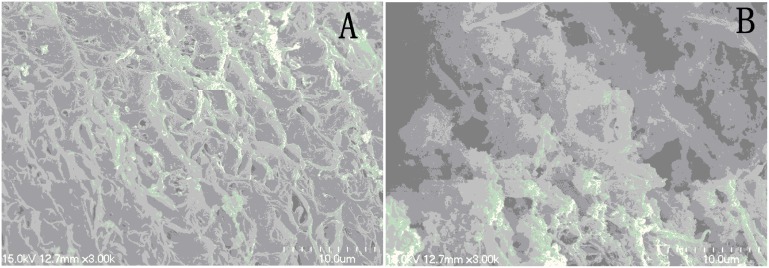
Scanning electron microscopy (SEM, ×3000) observation on male adult *Schistosoma japonicum* exposed to the medium containing DW-3-15 (25 µM, 17.4 g/mL) for 72 h. A: The mid-region of body wall shows fusion of ridges after incubation with DW-3-15*;* B: The inner wall of shows gynecophoral canal clefts after incubation with DW-3-15 for 72 h.

**Figure 5:**
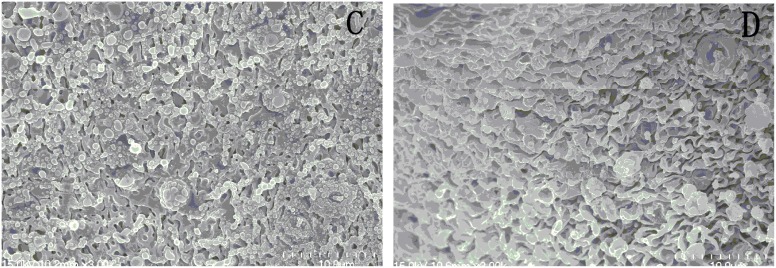
Scanning electron microscopy (SEM, ×3000) observation on male adult *Schistosoma japonicum* collected from mice 24 h after administration of DW-3-15 (200 mg/kg). C: The mid-region of body wall. D: The inner wall of the worm gynecophoral canal.

After treatment with 25 µM DW-3-15 for 72 h, the tegumental ridges became blurry, loosing of gynecophoral canal clefts, fusion of ridges and severe damage to the tegument were recorded (Figure 4A). Figure 5, C and D, shows the picture of the worm midbody portion as well as the gynecophoral canal of a male worm after treatment with DW-3-15 *in* *vivo*. While the damage is slight in contrast to that from the direct worm drug interaction *in* *vitro*, similar worm appearance was recorded and observed.

## Discussion

Combination of PZQ with artemether for the treatment of acute schistosomiasis has been noticed and tested for synergistic effects. Previous research from Shuhua et al observed no apparent efficacy increase when treated intragastrically by PZQ combined with artemether using infected mice mode [27]. Later on, a significant worm burden reduction was reported on infected rabbit mode [28]. The results from Utzinger’s studies using the combination regimen demonstrated significantly enhanced worm burden reduction compared to those achieved with either PZQ or artemether alon [29]. To further support the benefits of this regimen, the combination of PZQ and artesunate showed higher cure rate in patients compared to those who received either PZQ or artesunate alone in clinical trial [20]. However, the combination of PZQ and artesunate was not recommended in areas where both schistosome and malaria parasites coexist, for the concern that large-scale use of artemisinin derivatives might induce drug resistant parasites. Therefore, rational design of novel hybrid molecules of PZQ and ARTs with dual mode of actions would represent an attractive strategy in providing more effective antischisotomal agents with reduced risk of developing drug resistance.

The PZQ and artesunate conjugate DW-3-15 was developed from our laboratory, previous study has recorded potent worm killing effects *in* *vitro*, and a 56.2% worm burden reduction against adult stage of schistosomes at a single oral dose (200 mg/kg) using the infected mice mode [23]. Our current study further demonstrated that DW-3-15 exhibited superior worm reduction activity against *S. japonicum* at all developmental stage *in* *vivo*. Specifically, DW-3-15 was prepared by linkage of artesunate to PZQ through installing a hydroxyl group at the 10- position of PZQ, its activity against juvenile worm is comparable to that of artesunate but significantly improved compared to PZQ. More importantly, it retains the potency against the adult *S. japonicum* as PZQ with overall reduction rate being >60%, thus firmly support our design rationale.

Another series of hybrid drug trioxaquines have also been demonstrated to be active on both the larval and adult forms of the *Schistosoma mansoni* worms. The reported worm reduction rate discriminated greatly on schistosomula and adult worm, while the former is higher and the latter is significantly lower than that of PZ [30]. By comparison, DW-3-15 favors the high sensitivity against all worm developmental stage. Other hybrid compounds, for example, Yuxiang et al designed and synthesized an ozonide derivative of PZQ, were totally devoid of antischistosomal activit [22]. Patra M. et al have synthesized some ferrocenyl derivatives of PZ [15]. Again, compounds displayed only weak potency against *Schistosoma mansoni in* *vitro*, no *in* *vivo* worm killing activity was indicated. In all cases, the ozonide or the ferrocenyl moieties was structurally linked to PZQ through the cyclohexyl ring position. Therefore, it seems that linkage through the metabolically liable cyclohexyl could not afford active derivatives. Interestingly, other organometallic PZQ derivatives, the chromium tricarconyl moieties were covalently bond to the PZQ aromatic ring, were demonstrated to retain low worm burden reduction activit [16]. Apparently, chemical modification position played an important role on the compound activity. Indeed, modification based on PZQ would provide derivatives with broad antischistosomal activity.

The studies of DW-3-15 in mice harboring simultaneously with juvenile and adult schistosomes also showed promising results. At the early stage of reinfection, DW-3-15 exhibited comparable worm reduction activity to artesunate, and improved activity compared to PZQ. Our results suggested that DW-3-15 would serve as a new lead for further drug development. This is especially significant in areas where reinfection chances are high and retreatment with PZQ is necessary.

The dose-response study revealed that DW-3-15 at 100-mg/kg was not sufficient to achieve optimal worm reduction effect, only 40% adult worm reduction rate was observed. A dose of 400 mg/kg achieved the maximum effect, thus suggesting that the suitable dose for DW-3-15 is between 100–400 mg/kg under the current experimental conditions. Certainly, further experiments are warranted to identify the optimal dose for DW-3-15. We have also examined the toxicity of DW-3-15 using healthy mice and found that its LD_50_ is around 4.0 g/kg (unpublished-work), suggesting there is a broad therapeutic window for this compound.

The morphology observation from SEM image indicated that the potent anti-schistosomal activities of DW-3-15 might be correlated with its effects on worms tegument. The drug caused damage to both outside and inner wall of the worm tegument, suggesting the drug might exert similar action mechanism to PZQ. Likewise, mefloquine and trioxaquine PA1259 could also lead to severe tegument damage [30]. Consequently, the parasite was vulnerable to the host immune system because of the surface antigen exposure.

In conclusion, our results demonstrated that DW-3-15, a lead conjugate containing the complementary function moieties of PZQ and artesunate, induced severe damage to the worm tegument, thus leading to potent cytotoxic effects against both juvenile and adult stage schitosomes. Collectively, the biological evaluation results validated our hybrid antischistosomal drug design rational and strongly encourage further development of DW-3-15 as a new lead to design more potent analogs with novel dual mode of action.
